# Supplementary material for: Morphology of Vavilovia formosa (Steven) Fed. Nodules Induced by Different Rhizobia Strains
Source: Plants (Basel). 2025 Dec 10;14(24):3764. doi: 10.3390/plants14243764 (PMC12736739; doi:10.3390/plants14243764)
Supplement: Supplementary file 1 [file plants-14-03764-s001.zip › plants-3954756-supplementary.pdf]

Electronic Supplementary Material

Morphology of *Vavilovia formosa* (Steven) Fed. Nodules Induced by Different Rhizobia Strains

Anna V. Tsyganova [1*], Artemii P. Gorshkov [1], Anastasiia K. Kimeklis [1,2], Olga P. Onishchuk [1], Maxim G. Vorobiev[2], Evgeny E. Andronov [1] and Viktor E. Tsyganov [1]

[1] All-Russia Research Institute for Agricultural Microbiology, Saint Petersburg 196608, Russia; a.gorshkov@arriam.ru (A.P.G.), akimeklis@arriam.ru (A.K.K.), o.onishuk@arriam.ru (O.P.O), e.andronov@arriam.ru (E.E.A), vetsyganov@arriam.ru (V.E.T.)

[2] Saint Petersburg State University, 199034, Saint Petersburg, Russia; vorobiev.maxim@spbu.ru (M.G.V.)

*Author for correspondence:

Anna V. Tsyganova

Tel: +7 812 4705100

E-mail: avtsyganova@arriam.ru

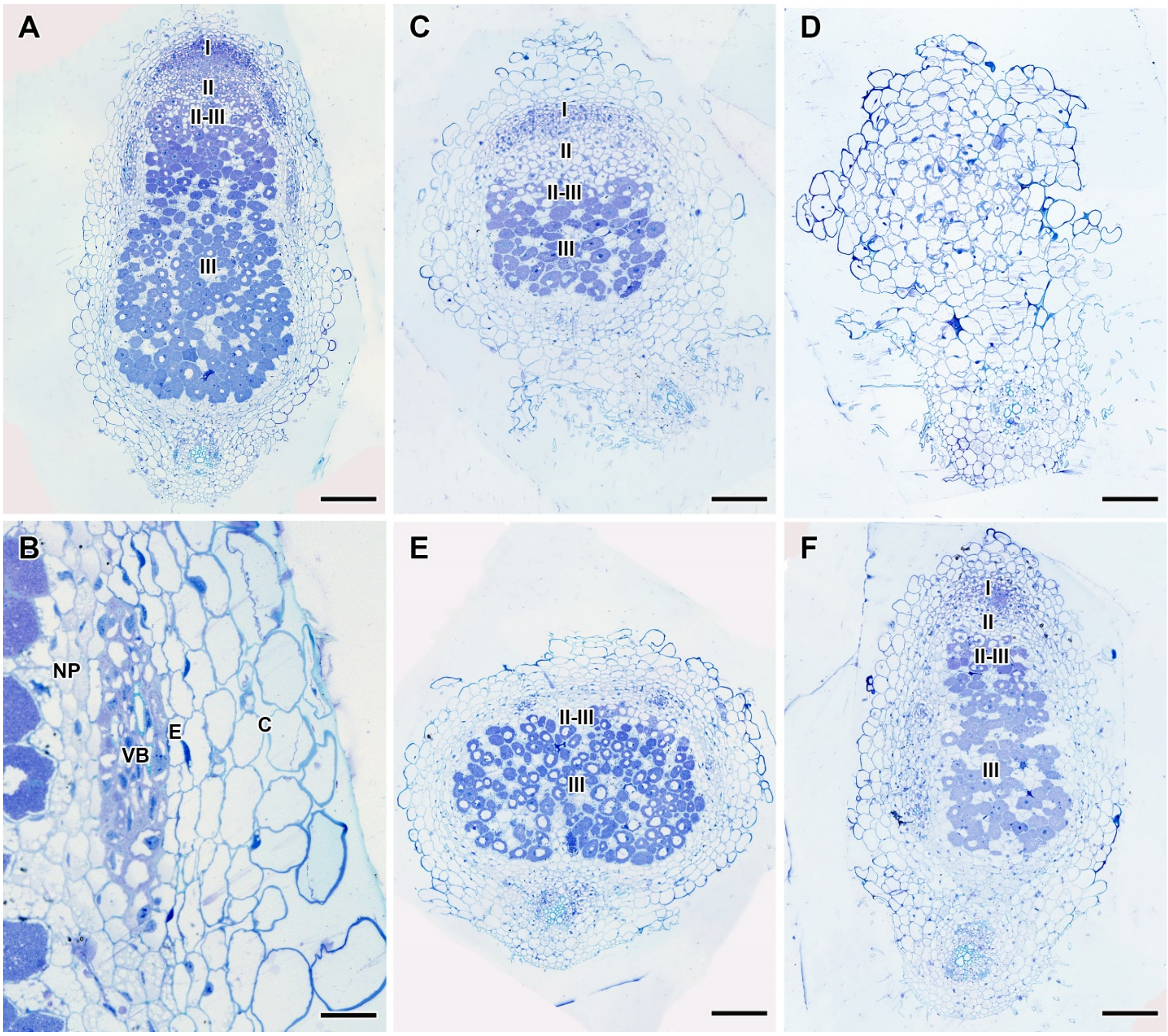

Figure S1. Histological organization of *Vavilovia formosa* (Steven) Fed. nodules induced by *Rhizobium leguminosarum* sv. *viciae* strains Vaf-12 (A, B), Vaf-108 (C, D), TOM (E), and *Rhizobium ruizarguesonis* strain RCAM1026 (F). (A, C–F) Longitudinal section of a nodule. (B) The structure of the peripheral zone in the nodule. Light microscopy, methylene blue-azur II staining. I, meristem; II, infection zone; II–III, interzone; III, nitrogen fixation zone; C, cortex; E, endodermis; NP, nodule parenchyma; VB, vascular bundle. Bars 200 μm (A, C, E, F), 50 μm (D); 10 μm (B).

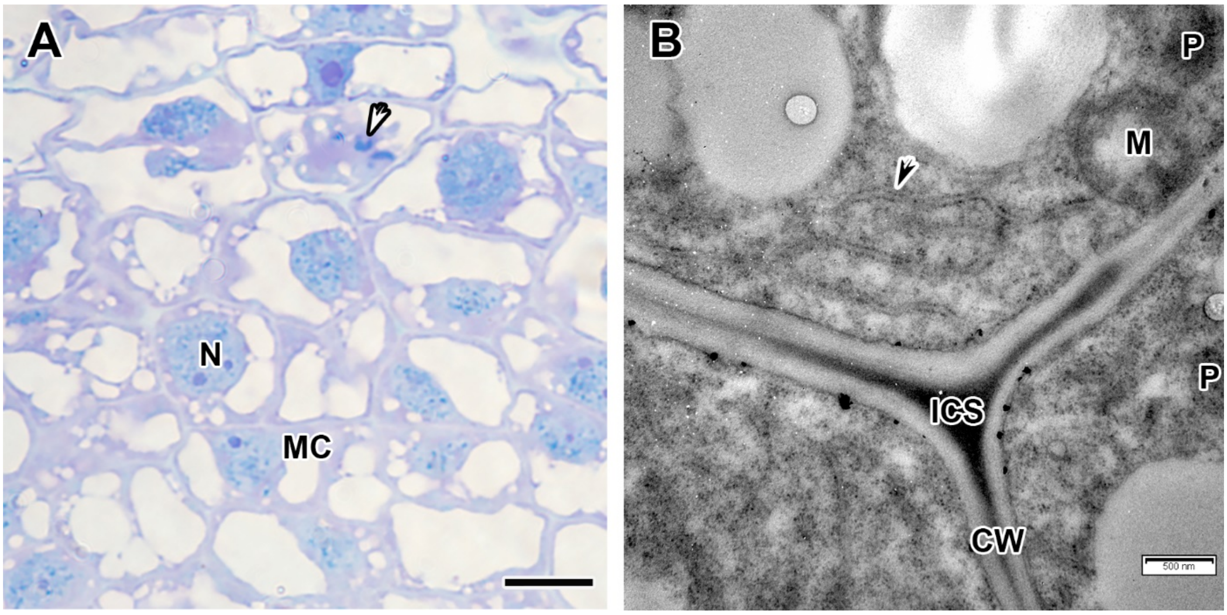

Figure S2. Meristem in the *Vavilovia formosa* (Steven) Fed. nodules induced by *Rhizobium ruizarguesonis* strain RCAM1026. (A) Histological organization. (B) Three-cell junction from the meristem. Light microscopy, methylene blue-azur II staining (A); transmission electron microscopy (B). CW, cell wall; ICS, intercellular space; M, mitochondrion; MC, meristematic cell; N, nucleus; P, proplastide; black arrowhead indicates endoplasmic reticulum profiles; white arrowhead indicates mitotic figure. Bars 5 μm (A), 500 nm (B).

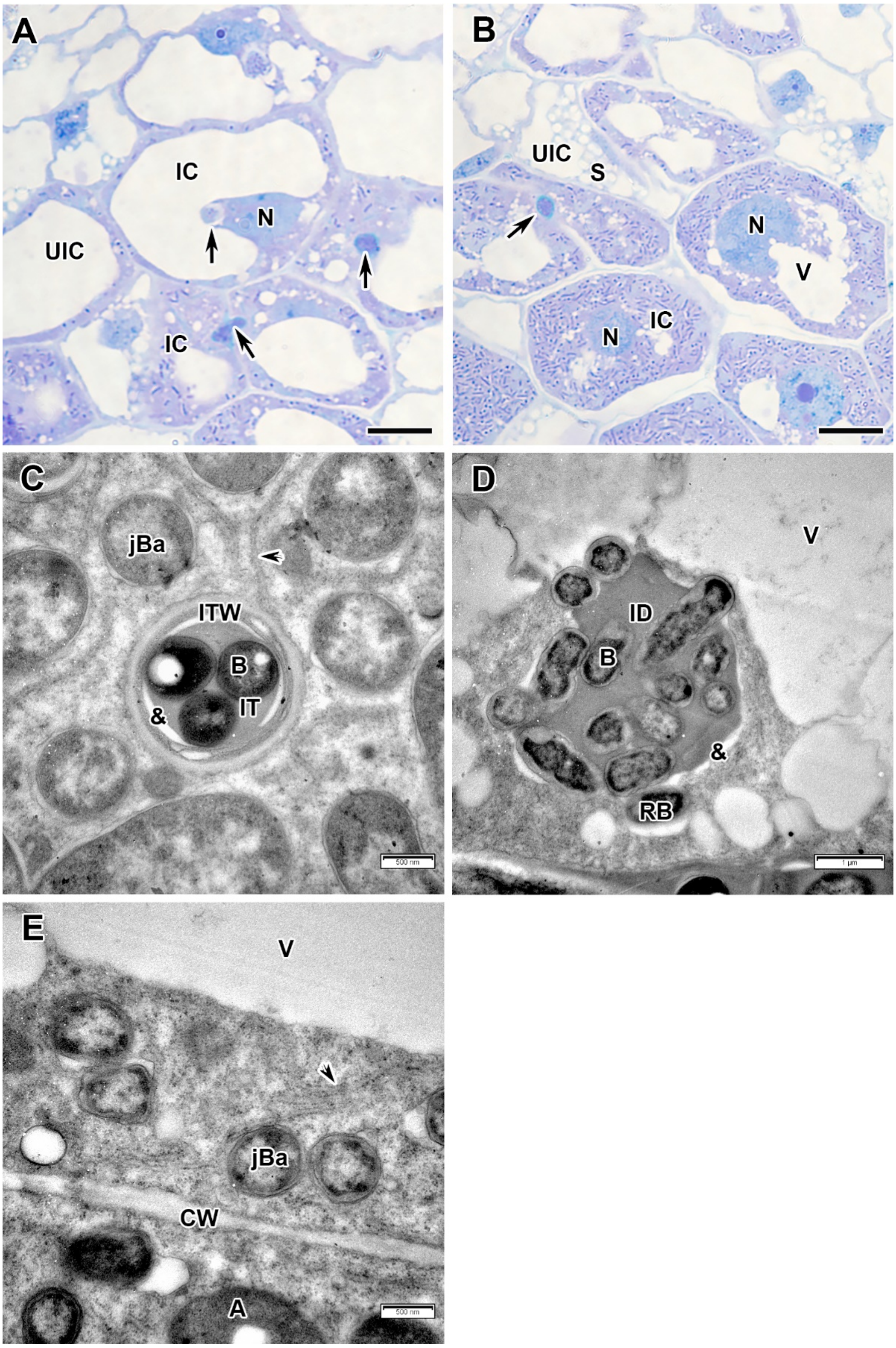

Figure S3. Infection zone in the *Vavilovia formosa* (Steven) Fed. nodules induced by *Rhizobium ruizarguesonis* strain RCAM1026. (A) Cells from the early infection zone. (B) Cells from the late infection zone. (C) An infection thread from the infection zone. (D) An infection droplet from the infection zone. (E) Juvenile bacteroids in the infected cell from the infection zone. Light microscopy, methylene blue-azur II staining (A, B); transmission electron microscopy (C–E). A, amyloplast; B, bacterium; CW, cell wall; IC, infected cell; ID, infection droplet; IT, infection thread; ITW, infection thread wall; jBa, juvenile bacteroid; N, nucleus; RB, releasing bacterium; S, starch grain; UIC, uninfected cell; V, vacuole; &, electron-transparent ring around infection thread and droplet matrix; black arrowheads indicate endoplasmic reticulum profiles, large black arrows indicate infection threads. Bars 5 μm (A), 2 μm (F), 1 μm (E), 500 nm (B–D).

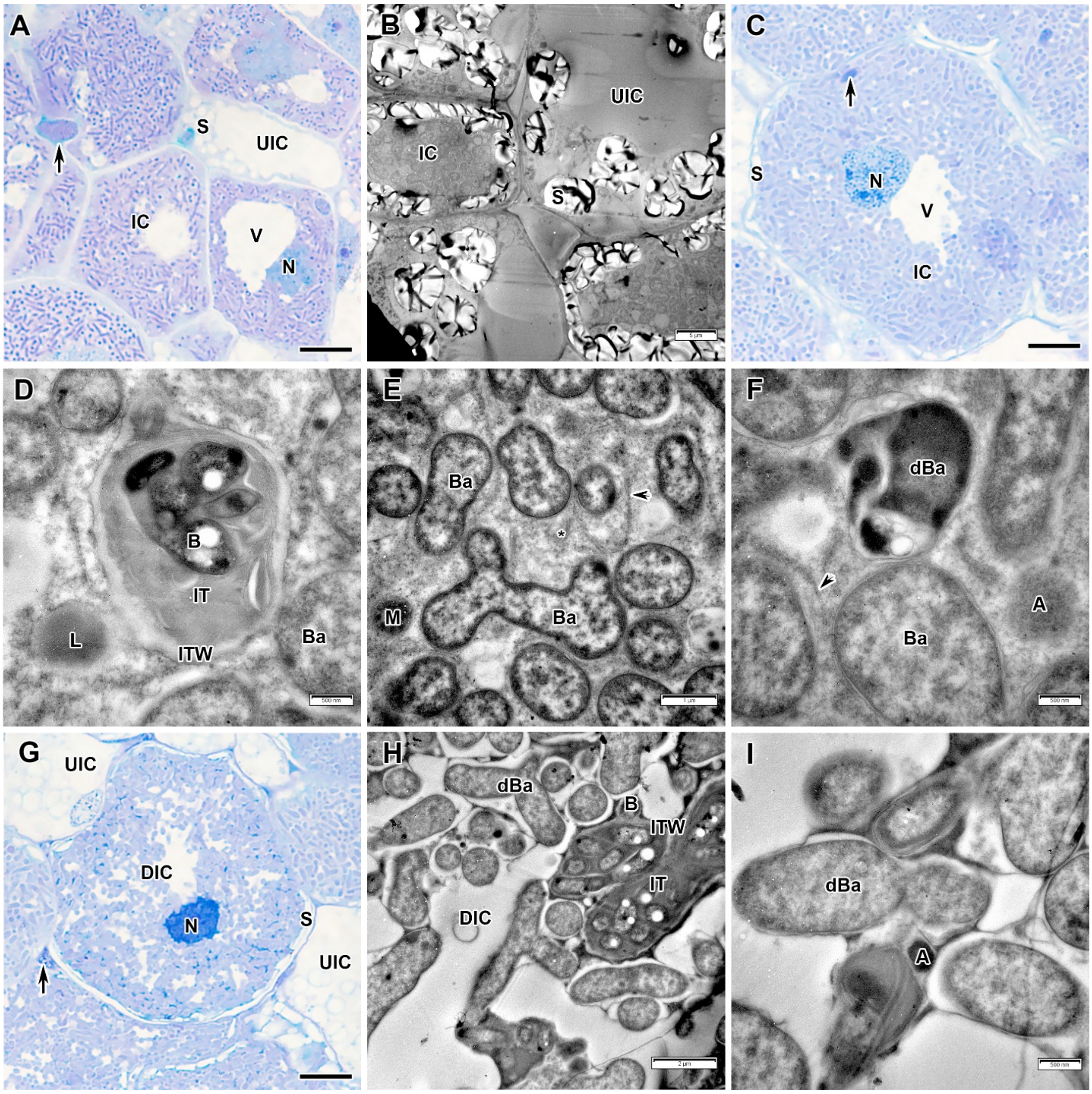

Figure S4. Interzone II–III and nitrogen fixation zone in the *Vavilovia formosa* (Steven) Fed. nodules induced by *Rhizobium ruizarguesonis* strain RCAM1026. (A) Cells from the interzone II–III. (B) Cells from the interzone II–III with starch accumulation. (C) An infected cell from the nitrogen fixation zone. (D) An infection thread from the nitrogen fixation zone. (E) Mature bacteroids from the nitrogen fixation zone. (F) Degenerating bacteroid from nitrogen fixation zone. (G) A degrading infected cell. (H) An infection thread in the degrading infected cell. (I) Degenerating bacteroids from the degrading infected cell. Light microscopy, methylene blue-azur II staining (A, C, G); transmission electron microscopy (B, D–F, H, I). A, amyloplast; B, bacterium; Ba, bacteroid; dBa, degenerating bacteroid; DIC, degrading infected cell; IC, infected cell; IT, infection thread; ITW, infection thread wall; L, lipid body; M, mitochondrion; S, starch grain; UIC, uninfected cell; V, vacuole; *, Golgi body; black arrowheads indicate endoplasmic reticulum profiles; large black arrows indicate infection threads. Bars 5 μm (A–C, G), 2 μm (H), 1 μm (E), 500 nm (D, F, I).
